# A clear cancer cell line (150057) derived from human endometrial carcinoma harbors two novel mutations

**DOI:** 10.1186/s12885-020-07567-w

**Published:** 2020-11-03

**Authors:** Yu-Hsun Chang, Dah-Ching Ding

**Affiliations:** 1 Department of Pediatrics, Hualien Tzu Chi Hospital, Buddhist Tzu Chi Foundation, and Tzu Chi University, Hualien, Taiwan; 2 Department of Obstetrics and Gynecology, Hualien Tzu Chi Hospital, Buddhist Tzu Chi Foundation, and Tzu Chi University, No. 707, Chung-Yang Rd., Sec. 3, Hualien, Taiwan, Republic of China; 3 Institute of Medical Sciences, Tzu Chi University, Hualien, Taiwan

**Keywords:** Clear cell carcinoma, Endometrium, Cell line, Proliferation, Mutation

## Abstract

**Background:**

Cell lines are extremely useful for both basic and clinical research. Thus, establishing endometrial cancer cell lines with malignant histology is important. This study aimed to extensively characterize an endometrial clear cell carcinoma cell line.

**Methods:**

This cell line, named 150,057, was derived from the endometrial clear cell cancer of a 63-year-old woman. The morphology, chromosomes, chemosensitivity, tumor markers, xenotransplantation characteristics, and cancer-related genes of the cell line were characterized.

**Results:**

This cell line exhibited adequate growth, being passaged more than 70 times. The morphology of the cells was polygonal with a cobblestone-like appearance. Karyotyping of the cell line revealed a hypodiploid chromosomal number. 150057 cells expressed CA19–9 and CA125. The cell line was sensitive to doxorubicin, paclitaxel, carboplatin, and cisplatin. After the cells were transplanted into the subcutaneous region of non-obese diabetic-severe combined immunodeficiency mice, they generated xenograft tumors with similar histology as the original tumor. A total of 59 somatic nucleotide mutations were identified in 25 of the 53 examined tumor suppressor genes and oncogenes. Two novel mutations were found in *FGFR3* and *ARID1A*.

**Conclusion:**

We established and characterized an endometrial clear cell carcinoma cell line that may be useful in carcinogenesis and treatment research for endometrial cancer.

**Supplementary Information:**

**Supplementary information** accompanies this paper at10.1186/s12885-020-07567-w.

## Background

Cancer is the second leading cause of death globally [1]. Endometrial cancer arising from the uterine endometrium is the most prevalent gynecologic cancer in Western countries [2]. The prognosis of endometrial cancer depends on various tumor characteristics determined through staging surgery [3]. Most patients with endometrial cancer are diagnosed with early-stage disease, and consequently, they have a fair prognosis. Nevertheless, if patients are diagnosed with late-stage endometrial cancer and lymph node metastasis, then treatment options are limited [3]. Thus, a comprehensive investigation of cancer biology is urgently required for endometrial cancer.

Histologically, endometrial cancer is divided into two categories [4]. Type 1 endometrial cancer exhibits endometrioid histology. It is low-grade cancer with a good prognosis. Type 2 endometrial cancer exhibits nonendometrioid histology. It is high-grade cancer with a poor prognosis. Clear cell cancer (CCC) of the endometrium is a rare endometrial cancer that comprises less than 3% of all cases of endometrial cancer [5, 6]. Endometrial CCC is a type 2 cancer with a generally poor prognosis [4]. However, endometrial cancer cannot be divided into only two types because of its heterogeneity [4]. The other two types of endometrial cancer, namely endometrioid and serous carcinoma, can be divided into four categories on the basis of somatic mutations, microsatellite instability, and copy number variation [7]. However, because of its rarity, the genomic landscape of endometrial CCC has not been clarified.

A unique cancer cell line is important for the progress of cancer research. It can be used to study cancer treatment mechanisms, dosage, toxicity, and other factors. According to previous reports, 24 endometrial cancer cell lines are available [8, 9], none of which is CCC. Many CCC cell lines from ovarian cancer exist [10]. Nevertheless, differences exist between ovarian and endometrial CCC [11].

Therefore, this study aimed to characterize a new endometrial CCC cell line derived from a patient with cancer.

## Methods

### Materials

The patient was a 63-year-old woman who was diagnosed with endometrial cancer several years ago. Her tumor marker levels were elevated (CA125, 70.2 IU/mL; CA199, 52.8 IU/mL). We performed a laparoscopic abdominal total hysterectomy, bilateral salpingo-oophorectomy, bilateral pelvic lymph node dissection, and omentectomy. Histology revealed mixed high-grade clear cell carcinoma and grade 1 endometrioid adenocarcinoma of the endometrium. The level of myometrial invasion was less than 50%. No regional lymph node metastasis or lymphovascular invasion was noted. The FIGO stage was IA. We obtained informed consent from the patient before surgery. The entire study protocol was approved by the institutional review board (Research Ethics Committee, Hualien Tzu Chi Hospital, IRB 101–09). We confirmed that all experiments were performed in accordance with relevant guidelines and regulations.

After surgery, she received adjuvant chemoradiation with cisplatin to treat high-grade CCC. After treatment, the tumor markers regressed to normal levels.

### Histology and immunohistochemistry of the tumor

We used 10% formalin (Sigma-Aldrich, St. Louis, MO, USA) to fix the tumor sections, followed by embedding in paraffin. Tissue was cut to a thickness of 4 μm and dewaxed in xylene for 5 min three times followed by 100% alcohol for 5 min, 90% alcohol for 5 min, and 80% alcohol for 5 min. The samples were then washed in PBS (Gibco) for 5 min. Tissues were blocked with hydrogen peroxide for 10 min, followed by three washes in PBS for 3 min each and UV block for 5 min. Samples were then washed with PBS three times for 3 min each. The sections were stained with H&E (Dako, Agilent, Santa Clara, CA, USA). Sections were also incubated with primary monoclonal antibodies at 4 °C overnight. Tissues were subsequently incubated with an HRP-linked secondary antibody for 10 min and then with diaminobenzidine tetrahydrochloride (DAB, Thermo Fisher Scientific, Waltham, MA, USA) for 5 min to detect reactivity. Slides were counterstained lightly with hematoxylin for 5 min, dehydrated, and mounted in mounting medium (Histokitt, Assistent, Altnau, Germany). We recorded photographs of the stained sections using a light microscope (Nikon TE2000-U fitted with a digital camera [Nikon DXM1200F], Nikon, Tokyo, Japan).

Antibodies against WT1 (1:200, GeneTex) [12], MIB1 (1:500, GeneTex) [13], Annexin IV (1:200, GeneTex) [14] were previously reported expressions in CCC, were utilized for identifying CCC. Antibodies against estrogen receptors (ER) (1:200, GeneTex), and progesterone receptors (PR) (1:200, GeneTex) were utilized to identify hormone receptors.

#### Western blot

Original and xenograft tumor tissue lysates were loaded onto a gradient 5–20% sodium dodecyl sulfate-polyacrylamide gradient gel. After electrophoretic separation, the proteins were transferred to a polyvinylidene difluoride membrane (Bio-Rad). The membrane was blocked at room temperature in a solution of 3% nonfat dry milk in PBS and 0.1% Tween-20 and then rinsed in PBS/0.1% Tween-20. Blots were incubated with diluted solutions of anti-P53 (1:1000, Cell Signaling Tech, Danvers, MA, USA) [15] and anti-HNF-1β (1:500, GeneTex) [16, 17] antibodies (previously reported expression in CCC) and treated with 1:5000 diluted anti-rabbit immunoglobulin G horseradish peroxidase (HRP) for staining (Amersham GE, Taipei, Taiwan). GAPDH proteins (1: 200, GeneTex) were used as internal controls. HRP signals were detected using an electrochemiluminescence kit (Promega, Fitchburg, WI, USA).

### Tumor cell culture conditions

The endometrial tumor (2 × 2 cm^2^) was finely cut into small pieces (1–2 mm^3^) using no. 15 blades in a 10-cm dish containing Dulbecco’s Modified Eagle’s Medium (DMEM, Gibco, Grand Island, NY, USA) without serum. The tissues were dissociated with 0.1 mg of collagenase IA (Sigma-Aldrich) and incubated for 60 min at 37 °C. After enzymatic digestion, the resultant cells were placed in DMEM (Invitrogen-Gibco, Carlsbad, CA, USA) supplemented with 10% fetal bovine serum (Biological Industries, Kibbutz, Israel), penicillin, and streptomycin (Gibco). The cells passaged every week.

This cell line has been authenticated by the Center for Genomic Medicine, National Cheng Kung University, Taiwan after comparisons with ATCC profiles of short tandem repeats (STRs, www.atcc.org/STR%20Database.aspx) (Supplement Table 1). The common STR markers (TH01, D5S818, D13S317, D7S820, D16S539, CSF1PO, AMEL, vWA, and TPOX) were used to identify the cell line.

### The morphology of the cancer cells

We used a phase-contrast microscope (Nikon) to exam the morphology of cancer cells in a culture dish.

### Proliferation assay

Proliferation assays were performed at P10, P26, and P41. We placed cells in 96-well plates (Costar, Corning, Corning, NY, USA) at a density of 3000 cells/cm^2^ on day 1. The culture medium was changed every 3 days. We harvested the cells on days 7. Doubling time was determined using the equation doubling time = duration × log (2)/log (final cell number) − log (initial cell number) [18].

### The analysis of chromosomes

Aneuploidy is a unique feature of cancer cells [19]. To clarify the karyotypes of 150,057 cells, karyotyping of 150,057 cells was conducted at the Cytogenetics Laboratory of the Genetics Consultation Center, Hualien Tzu Chi Hospital. Briefly, cells were cultured to exponential growth and incubated with colchicine (Sigma) to arrest cells at metaphase. Cells were then exposed to a hypotonic solution to induce bursting. After bursting, cells were fixed on a glass slide and stained with Giemsa stain. One cytogeneticist reviewed chromosomes that were organized in karyograms. The chromosome number distribution was obtained after counting for 50 metaphases. The karyotypes of 50 metaphases were analyzed. The results of the chromosomes were reported in line with the 2016 International System for Human Cytogenetic Nomenclature.

### Immunofluorescence

Cells were fixed with 4% paraformaldehyde (Sigma) for 10 min, permeabilized with 0.1% Triton X-100 (Sigma) for 10 min, blocked with 4% normal goat serum (Sigma) for 30 min, and then treated with primary antibodies against CD133 (Miltenyi Biotec, Bergisch Gladbach, Germany) and CK7 (Bioss Inc., Woburn, MA, USA) overnight at 4 °C. Cells were then incubated with fluorescein isothiocyanate (FITC, goat anti-rabbit IgG antibody [DyLight488], GeneTex) or rhodamine (goat anti-mouse IgG antibody [DyLight594], GeneTex) at a concentration of 1:200 as the secondary antibody for 1 h at room temperature in the dark. Cell nuclei were stained with 4′,6-diamidino-2-phenylindole (1:200, Sigma). The coverslip was mounted with a drop of mounting medium (Histokitt, Assistent, Altnau, Germany), and cells were observed under a microscope.

### Flow cytometry

We used flow cytometry to quantify the proportion of CSCs in 150,057 cells. We detached the cells using PBS containing Accutase (Interchim, Montluçon, France). Then, we collected the cell pellet after centrifugation at 1200 rpm for 5 min. Cells were then washed three times with PBS. Thereafter, we incubated the cells with the target antibodies (CD133 [1:200, Miltenyi Biotec], CD326 [1:200, Miltenyi Biotec], and CK& [1:200, Bioss Inc]) conjugated with FITC or phycoerythrin (BD, PharMingen, Franklin Lakes, NJ, USA) for 30 min. After washing cells three times with PBS, cells were analyzed using a flow cytometer (Becton Dickinson, San Jose, CA, USA).

#### Elisa

ELISA was performed to assess tumor marker expression. In total, 2 × 10^6^ cells/5 mL were cultured for 7 days to examine tumor markers including CA125, CA 199, Carcinoembryonic antigen (CEA), HCG, and squamous cell carcinoma antigen (SCC) antigen. All ELISA kits were purchased from Thermo Fisher Scientific.

### Chemosensitivity assays

The current chemotherapy regimen for CCC includes doxorubicin, carboplatin, cisplatin, and paclitaxel [20]. The effects of doxorubicin (Adriblastina, Pfizer, Kent, NJ, USA), carboplatin (Abiplatin, ABIC Ltd., Netanya, Israel), cisplatin (CDDP, Bristol-Myers, New York, NY, USA), and paclitaxel (Formoxol, Yung Shin Pharm. Ind., Co. Ltd., Taichung, Taiwan) on tumor cells were compared. The 2H-tetrazolium, 2,3-bis (2-methoxy-4-nitro-5-sulfophenyl)-5-[(phenylamino)carbonyl]-hydroxide (XTT, Biological Industries Ltd.) assay was used to measure cell proliferation. Each well of 96-well plates contained 100 μL of medium and 5000 cells. After 48 h of incubation, various concentrations of drugs in 50 μL of medium were added for chemosensitivity experiments. Drugs were mixed with the XTT solution immediately before use. Specifically, 50 μL of XTT/N-methyl dibenzopyrazine methyl sulfate (Biological Industries Ltd) were added in 100 μL of culture medium. The optical density (OD) in each well was determined after 2–5 h of incubation at 37 °C. A spectrophotometer (ELISA reader, Dynex Technologies, Chantilly, VA, USA) was used to detect the OD at a wavelength of 450 nm (reference wavelength: 650 nm). Then, the IC50 of each drug in 150,057 cells was obtained. We used the 4PL method as described previously [21]. The equation is as follows: +d, where Y is the response, X is the concentration, a is the bottom of the curve, d is the top of the curve, b is the slope factor, and c is the concentration corresponding to the response midway between a and d. The experiments were done thrice.

### Xenotransplantation

Three female 6-week-old NOD-SCID mice (NOD.CB17-Prkdcscid/JTcu, median weight 20 g) were obtained from Tzu Chi University and inoculated with 1.5 × 10^5^ 150,057 CD133+ (P10) cells subcutaneously into their backs. We raised these mice in a pathogen-free room at a temperature of 22 °C and a relative humidity of 30–70% at the animal center in Tzu Chi University. The personnel in the animal center were in charge of their feeding. The development of a tumor was confirmed, and the tumor size and weight were measured. After 12 weeks, at which point the tumor reached 5–10 mm in diameter, the mice were euthanized with CO2 and followed by cervical dislocation. Then the tumor was removed. Each xenograft was processed for histological examination. The tumor slices were fixed in 10% formalin and embedded in paraffin. H&E staining was used to observe tumor morphology. Immunohistochemistry was used to observe specific protein expression. The Hualien Tzu Chi Hospital Animal Use Protocol Board provided full approval for this research (No. 104–05-01).

### Immunohistochemical staining of xenograft tumors

To assess specific cancer-associated protein expression, we examined WT1, MIB1, Annexin IV, ER, and PR in xenografts. The specimens in the paraffin block were cut to a thickness of 4 μm and deparaffinized. Then, the slices were stained with antibodies (P53, WT1, HNF1β, Annexin IV, ER, and PR, 1:200; MIB1, 1:500, all purchased from GeneTex) as described previously. DAB was then used to detect reactivity. We captured the images using a light microscope (Nikon).

### Mutational analysis

To perform mutational analysis, we extracted genomic DNA from 150,057 cells (P24) using a Qiagen kit (Qiagen, Hilden, Germany) following the manufacturer’s instructions. After the quality and quantity assessment, NGS was performed according to the instructions of Illumina (TruSeq Enrichment guide) [22]. Briefly, genomic DNA was fragmented, denatured, and hybridized. We utilized the Ion AmpliSeq™ Cancer Hotspot Panel v2 with 50 commonly oncogenes and tumor suppressor genes (Ion Torrent, Life Technologies, USA) as well as *ARID1A*, *CCNE1*, and *PPP2R1B* [23–27]. The captured sequences were then enriched and further amplified before being subjected to Illumina sequencing. Variant caller software was used for variant detection. Somatic nucleotide mutations were identified through the following filtering steps: (1) variant allele frequencies of > 30% and (2) sequencing coverage of > 40 reads.

## Results

### Histology of the tumor specimen

**Fig. 1:**
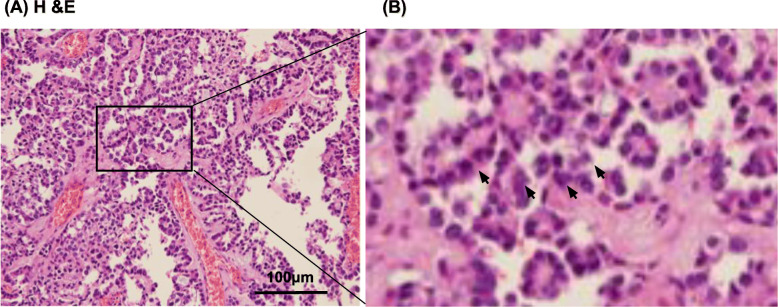
Histology of the primary tumor. **a** Hematoxylin and eosin staining revealed endometrial clear cell carcinoma. **b** Magnified view of the part of (**a**) A hobnail shape and clear cytoplasm (arrows) was noted. Scale bar = 100 μm

A tumor was excised from a 63-year-old woman with endometrial cancer. To clarify the histology expression status of the primary tumor, we stained the specimen with H & E staining. The original tumor was mixed endometrioid and clear cell carcinoma (clear cytoplasm, Fig. 1a), and it had a hobnail shape (Fig. 1b).

### Establishment and characterization of a cell line

**Fig. 2:**
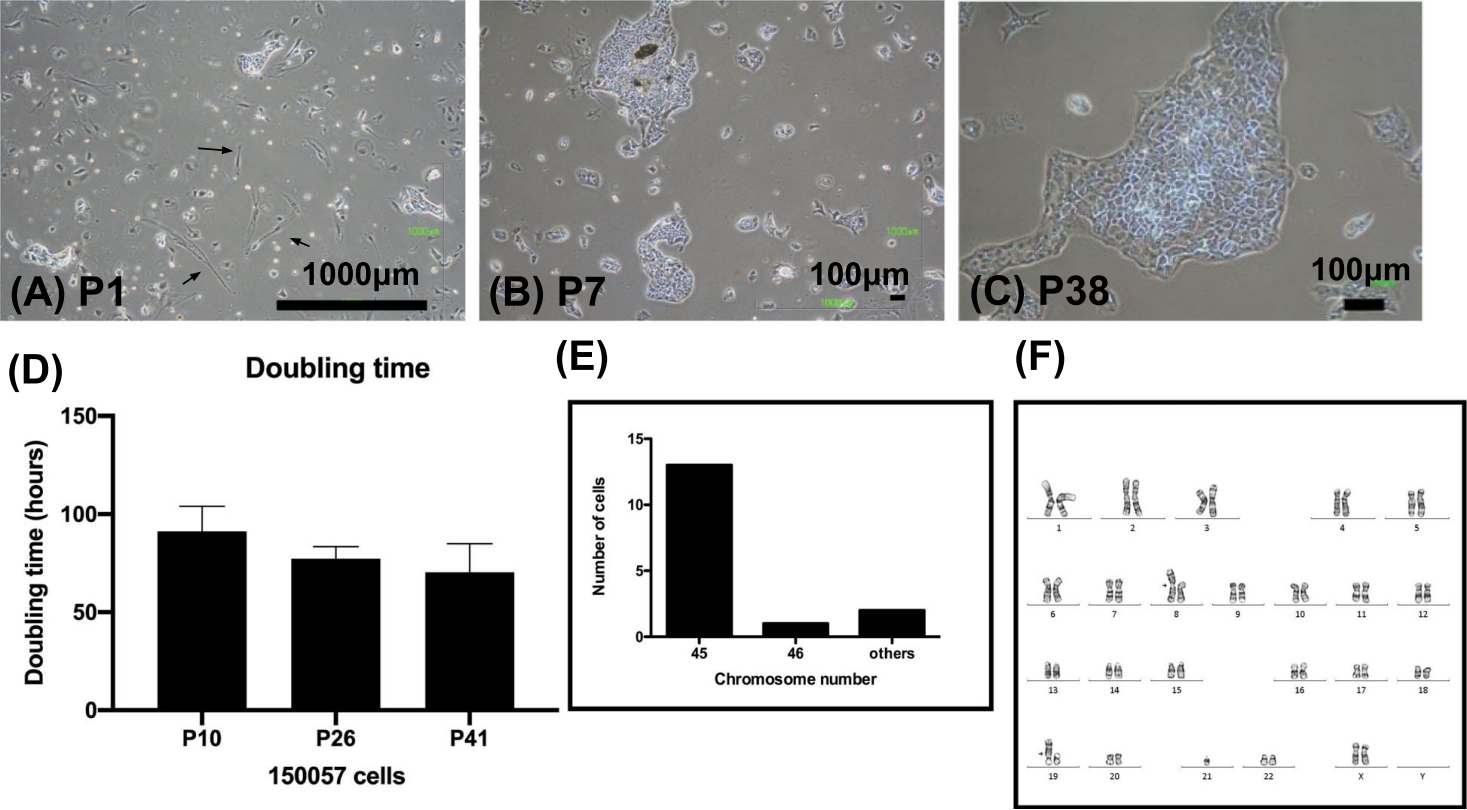
Morphology, growth curve and chromosome of 150,057 cells. Pavement-like arrangement in cells at (**a**) passage 1 (P1), (**b**) P7, and (**c**) P38. Arrows denote fibroblast-like cells. Scale bar = 1000 μm (**a**) or 100 μm (**b–c**). **d** Doubling time (mean ± standard deviation) of 150,057 cells at P10, P26, and P41 (*n* = 3 each). (**e**) The chromosome pattern exhibited a hypodiploid distribution. **f** The analysis of chromosomal number revealed abnormalities

Next, to clarify the characteristics of endometrial CCC, we cultured tumor cells isolated from the original tumor. The collagenase-dissociated cells developed distinct outgrowths after a 1-week culture period. Initially, a few fibroblasts with a spindle-like appearance were present (Fig. 2a). After serial passaging the cells, the fibroblasts disappeared (Fig. 2b–c), being replaced by epithelial-like cells with a pavement-like arrangement and polygonal shape (Fig. 2b [passage 7 (P7)] and 2C [P38]). The doubling time was examined at P10, P26, and P41 of the 150,057 cell line. The doubling times were no statistical difference among cells at P10 (91.7 ± 12.7 h), P26 (76.2 ± 6.7 h), and P41 (70.4 ± 14.0 h) (Fig. 2d). Over 70 serial passages were successively conducted. The cells continued to display continual stable growth even after the study was completed. The cell line authentication results confirmed the presence of 150,057 cells without cross-contamination by any ATCC cell line (Supplement Table 1).

### Tumor-derived cells exhibited aberrant chromosomes

Chromosomes are often aberrated in solid tumors. Therefore, in this study, we analyzed the chromosome pattern in the tumor cells. The chromosome number was hypodiploid (Fig. 2e). Chromosomal analysis revealed the following abnormalities: 45,XX,idic (8)(p11.2),der (19) t (8:19)(q11.2;p13.3),-21 [10]/44 ~ 46,XX,idic (8)(p11.2),der (10) t (10:17)(p12;q11.2),der (19) t (8,19)(q11.2;p13.3),-21[cp5] (Fig. 2f).

### Tumor-derived cells expressed CD133 and EpCAM

Cancer stem cells (CSC) can cause tumor chemoresistance and recurrence. Therefore, we next identified the CSC population in 150,057 cells using immunofluorescence and flow cytometry. CD133 was used as a CSC marker, and EPCAM and cytokeratin 7 (CK7) were used as epithelial cell markers.

**Fig. 3:**
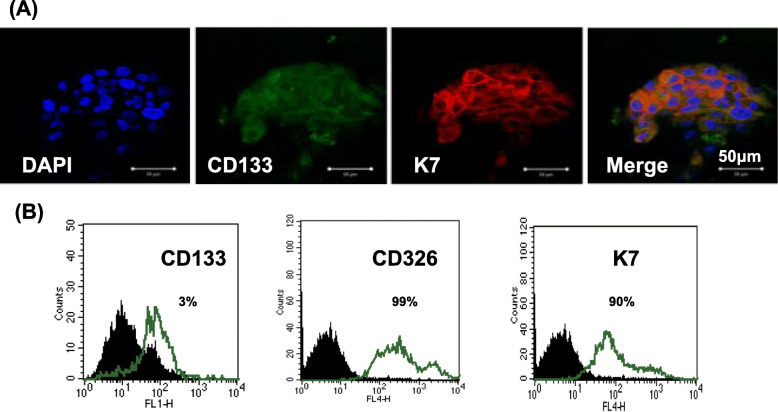
Fluorescence immunohistochemistry and flow cytometry of 150,057 cells. **a** Immunohistochemistry revealed positive staining for CD133 and cytokeratin-7 (CK7). 4′,6-Diamidino-2-phenylindole staining (blue) revealed the locations of nuclei. **b** Flow cytometry revealed that 150,057 cells were positive for CD326 and CK7. CD133+ cells comprised only 3% of the cell population

Immunofluorescence revealed positive staining for CD133 and CK7 (a mullerian origin marker) (Fig. 3a).

**Fig. 4:**
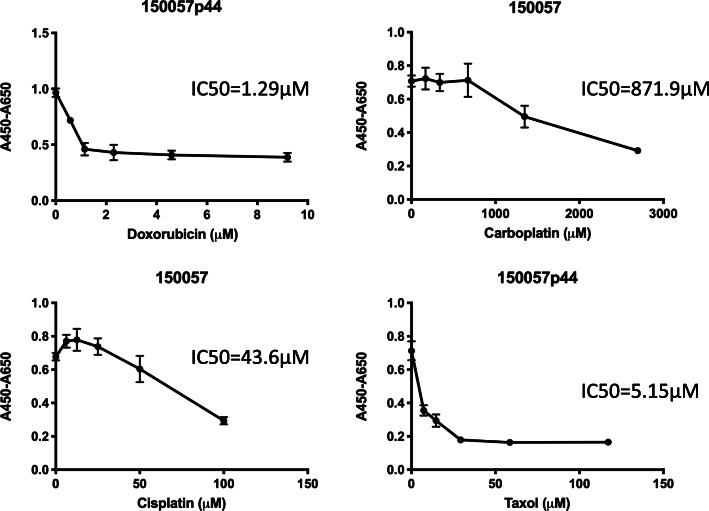
In vitro chemosensitivity of 150,057 cells. 150,057 cells were cultured with various concentrations of various chemotherapeutics (doxorubicin [**a**], carboplatin [**b**], cisplatin [**c**], and paclitaxel [**d**]) for 48 h. The 2H-tetrazolium, 2,3-bis (2-methoxy-4-nitro-5-sulfophenyl)-5-[(phenylamino)carbonyl]-hydroxide assay was used to quantify cell proliferation. We also calculated the half-maximal inhibitory concentration (IC50, μM, mean ± standard deviation, *n* = 3 each) of each drug

Flow cytometry indicated that only 3% of the tumor cells expressed CD133 (Fig. 4b). Flow cytometry also revealed that 99% of the tumor cells expressed EpCAM (CD326, a marker of breast and colon CSCs, Fig. 3b).

### Chemosensitivity

To assess the drug sensitivity of 150,057 cells, we exposed them to four commonly used chemotherapeutics in endometrial cancer, namely doxorubicin, carboplatin, cisplatin, and paclitaxel. The half-maximal inhibitory concentrations (IC50s) in 150,057 cells were 0.7 μg/mL for doxorubicin (Fig. 4a), 323.7 μg/mL for carboplatin (Fig. 4b), 13.1 μg/mL for cisplatin (Fig. 4c), and 4.4 μg/mL for paclitaxel (Fig. 4d).

### 150,057 cells exhibited increased CA125 and CA19–9 expression

To determine tumor marker expression in the 150,057 cells, we collected conditioned medium from 150,057 cells and used Enzyme-linked immunosorbent assay (ELISA) to evaluate tumor marker levels. Positivity was noted for CA125 (1091.6 U/mL) and CA19–9 (65.9 U/mL), whereas CEA, human chorionic gonadotropin (HCG), and SCC antigen expression was not detected (Table 1). The increased expression of CA125 and CA199 in 150,057 cells was compatible with the findings in the primary tumor.

**Table 1:** The concentration of tumor markers in the conditioned medium of 150,057 cells

Tumor marker	Mean	Standard deviation
CEA (ng/ml)	0.85	0.1
HCG (mIU/ml)	8.4	0.6
SCC (ng/ml)	0.5	0
CA125 (U/mL)	1091.6	16.8
CA199 (U/mL)	65.9	1.4

*CEA* Carcinoembryonic antigen, *HCG* Human chorionic gonadotropin, *SCC* Squamous Cell Carcinoma antigen, *CA125* Cancer antigen 125, *CA199* Carbohydrate antigen 199

### CD133+ tumor cells could generate xenograft tumors

**Fig. 5:**
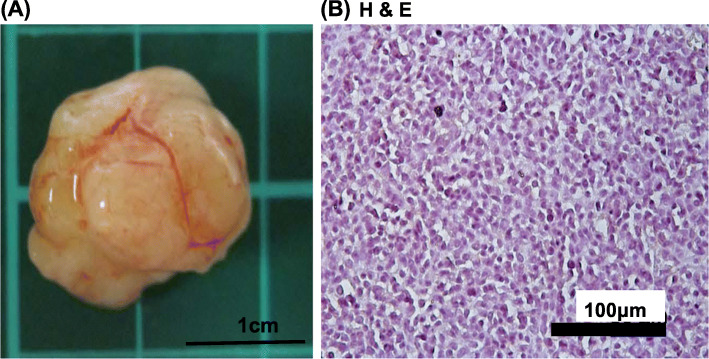
Histology of xenografted tumor. **a** The gross appearance of xenografted tumors (2 × 1 cm^2^ in size) formed in non-obese diabetic-severe combined immunodeficiency mice harboring CD133+ 150,057 cells. Scale bar = 1 cm. **b** Hematoxylin and eosin staining of the formed tumors revealed undifferentiated cancer. Scale bar = 100 μm

Xenografts are useful for assessing cancer development and treatment. In this study, we used non-obese diabetic-severe combined immunodeficiency (NOD-SCID) mice as a xenograft model for 150,057 cells. The results illustrated that xenografts were generated in all three examined mice after the injection of CD133+ 150,057 cells, and the average tumor size was approximately 2 × 1 cm^2^ (Fig. 5a). Hematoxylin and eosin (H&E) staining revealed the presence of undifferentiated endometrial cancer (Fig. 5b).

### Immunohistochemistry of the original and xenografted tumor

**Fig. 6:**
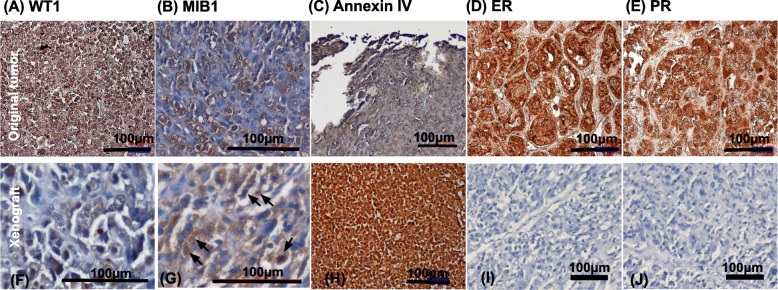
Immunohistochemical staining of the original and xenotransplanted tumor. Original and xenografted tumor cells were positive for WT1 (**a, f**), MIB1 (**b, g**) (arrows: nuclear staining), Annexin IV (**c, h**). However, estrogen receptor (ER) (**d, i**) and progesterone receptor (PR) (**e, j**) are only positive staining in the original tumor. Scale bar = 100 μm

Immunohistochemistry of the original and xenografted tumor revealed positivity for WT1 (Fig. 6a, f), MIB1 (Fig. 6b, g) (arrows: nuclear staining), Annexin IV (Fig. 6c, h). However, ER (Fig. 6d), and PR (Fig. 6e) were positive in the original tumor, but not detected in xenotransplanted tumors (ER: 6I, PR: 6 J).

### Protein expression of the original and xenografted tumor

**Fig. 7:**
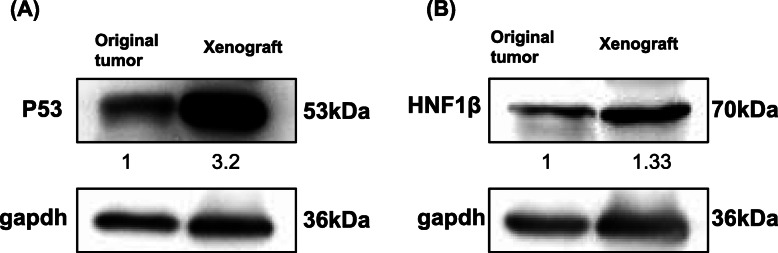
Western blot analysis of the original and xenotransplanted tumor. Original tumors and xenograft were positive for P53 **a** and positive for HNF1β (**b**). GAPDH was used as internal control. Numbers in the figure are represented as relative quantification to GAPDH. All cropped blots were run under the same experimental conditions. The full-length blots are presented in Supplementary Figure [7]

We used Western blot to detect another two proteins P53 and HNF1β which will be expressed in endometrial CCC to avoid non-specific binding of the proteins in immunohistochemistry. Figure 7 illustrated P53 and HNF1β expression in the original tumor and xenografted tumor. Original tumor and xenograft showed positive for P53 (Fig. 7a) and HNF1β (Fig. 7b).

### Mutational analysis

**Fig. 8:**
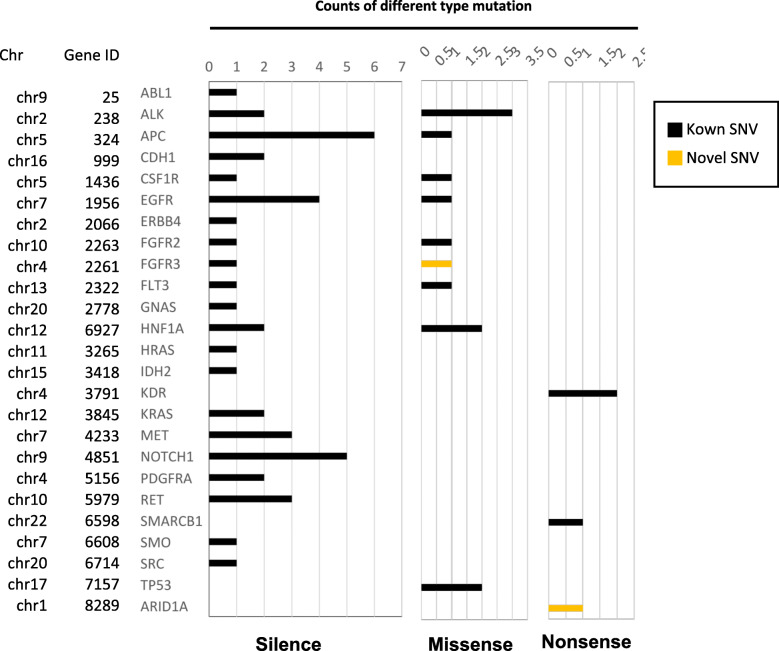
A total of 59 somatic nucleotide mutations were identified in 25 of 53 tumor-related genes in the 150,057 cell line. Two novel mutations were found in *FGFR3* and *ARID1A*. SNV: single nucleotide variant

Mutations in genes such as *TP53* and *PIK3R1* in endometrial cancer are correlated with tumor staging and survival [28]. Through next-generation sequencing (NGS) and analysis, we identified 59 somatic nucleotide mutations in 25 of the 53 examined tumor-related genes (Fig. 8), including 4 nonsense, 13 missense, and 42 synonymous substitutions. Among them, two nonsynonymous mutations in *FGFR3* and *ARID1A* were not reported in dbSNP and Catalog of Somatic Mutations in Cancer database (Table 2) [29]. Hence, they were identified as novel somatic mutations. Their gene products may alter physiological functions based on their SIFT scores < 0.05, indicating that they are deleterious.

**Table 2:** Two novel variants that have not been reported in the dbSNP and COSMIC database

	Chr	POS	Ref Allele	Variant	Genotype	Variant type	Allele source	Gene.refgene	ExonicFunc.refgene	AA ref	AA variant
1	chr4	1,803,663	G	A	het	SNP	Novel	FGFR3	nonsynonymous SNV	A	T
2	chr1	27,100,192	C	T	het	SNP	Novel	ARID1A	stop gain	Q	X

*chr* chromosome, *POS* position, *Ref* Reference, *het* heterogenous, *SNP* Single Nucleotide Polymorphism, *dbSNP* Single Nucleotide Polymorphism Database, *COSMIC* the Catalogue Of Somatic Mutations In Cancer, *SNV* single nucleotide variant, *AA* amino acid

## Discussion

In this study, we isolated and characterized an endometrial CCC cell line, which we named 150,057. Previous studies established several ovarian CCC cell lines [10, 30]. However, no endometrial CCC cell line had been previously established. A few differences have been observed between endometrial and ovarian CCC [11]. From the pathological perspective, the ovarian CCC comprises glycogen-containing clear cells and hobnail cells. Endometrial CCC is characterized as a type 2 cancer. However, endometrial CCC differs from serous carcinoma by the presence of mutations in *PTEN* and *ARID1A* and microsatellite instability [31].

Guidelines for the treatment of endometrial CCC have been published [32]. Surgical treatment is performed via staging surgery, which includes hysterectomy, bilateral salpingo-oophorectomy, and lymphadenectomy. If the pathological examination reveals an unfavorable type of cancer such as CCC, then adjuvant chemoradiation could be administered. The current chemotherapy regimen includes doxorubicin, carboplatin, cisplatin, and paclitaxel [20]. Our study also demonstrated that the derived cell line was sensitive to all these four chemo drugs. In clinical use, the carboplatin dosage is usually determined using the area under the curve and creatinine clearance (CCr). In gynecologic cancer, the dosage of carboplatin (mg) is calculated using the formula (CCr + 25) × AUC [33]. Therefore, the dosage of carboplatin clinical exceeds those of other chemotherapy drugs, in line with our in vitro findings.

Endometrial CCC shares some histopathological characteristics with ovarian CCC. Endometrial CCC expresses CK7, whereas CK20 expression is absent [34]. Endometrial CCC also exhibits low or negative expression for ER and PR [35]. Similarly, as ovarian CCC, endometrial CCC also expresses HNF1β, as do some endometrioid and serous cancers [36]. However, our original tumor was positive for ER and PR which may explain the good therapeutic response in our patient. Nevertheless, in 150,057 cells, the expression of ER and PR was reversed. This may have been caused by long-term culture, which may cause the loss of ER and PR expression [37].

The molecular biology of endometrial CCC is less clear than that of ovarian CCC. Ovarian CCC is typically *TP53* wild-type, and it exhibits little chromosomal instability. However, we found two point mutation of *TP53* gene in endometrial CCC. They were missense mutations that caused aberrant TP53 protein expression which may cause tumor growth and anti-apoptosis [38].

A high frequency of mutations is observed in *ARID1A* and *PIK3CA* [39, 40]. In endometrial CCC, BAF250a expression (encoded by *ARID1A*) is depleted [31]. Mutation of *ARID1A* causes chromatin remodeling dysfunction, which changes the expression of multiple genes including *CDKN1A*, *SMAD3*, *MLH1*, and *PIK3IP1*, thereby contributing to carcinogenesis [41]. These changes may be related to the pathogenesis of endometrial CCC. In our study, we found a novel mutation in the *ARID1A* gene in endometrial CCC.

ARID1A is a subunit of the switch/sucrose non-fermentable (SWI/SNF) complex, which is related to cell cycling and proliferation [42]. The SWI/SNF complex can change the DNA conformation, recruit transcription factors for DNA repair, proliferation, and replication [43]. Regarding ARID1A gene mutation in relation to response to therapy, only case reports and one phase 2 clinical trial have been reported [41]. Temsirolimus, a small-molecule inhibitor of the PI3K/AKT pathway, combined with trabectedin exhibited efficacy in a subset of patients with ovarian CCC featuring *ARID1A* mutation and PI3K/AKT pathway activation [41]. At present, a phase 2 clinical trial is examining temsirolimus in combination with trabectedin for ovarian CCC treatment [44]. The multikinase inhibitor sorafenib induced a partial response in a patient with stage IIIC ovarian CCC with *PIK3CA* mutation and resistance to mTOR inhibitors [41]. EZH2-targeting drug GSK126 can inhibit the proliferation of *ARID1A*-mutated ovarian CCC cells [45]. Taken together, *ARID1A* may represent a new target for the treatment of endometrial CCC.

FGFR3 is one of the 4 FGFR tyrosine kinases (FGFR1–4) [46]. When FGFR binding to its ligand, the tyrosine kinase will undergo phosphorylation and proceed the four main signaling pathways (MAPK, PI3K/AKT, PLCγ, and STAT) to activate antiapoptosis, cell growth, and proliferation [47]. FGFR3 gene mutation has never been reported in endometrial CCC. *FGFR3* mutation has been found in two-thirds of low-grade papillary bladder cancers [48]. *FGFR3-TACC3* fusion (potent oncogenes) has been reported to result in a related metabolic disturbance in glioblastoma and gastric cancer. *FGFR3* mutation is also correlated with poor prognosis in oral cancer [49]. *FGFR3* mRNA overexpression is linked to poor prognosis in colon cancer [50]. In our study, we also found a novel mutation of the FGFR3 gene in endometrial CCC.

Regarding *FGFR3* mutation in relation to therapeutic response, a previous study illustrated that the *FGFR3*-targeting drug anlotinib has better treatment efficacy in endometrial cancer than conventional chemotherapy with carboplatin and paclitaxel [51]. A phase 2 trial of nintedanib in patients with *FGFR*-mutated endometrial cancer reported an overall response rate (ORR) of 9.4%, a partial response rate of 9.4%, progression-free survival (PFS) of 3.3 months, and overall survival (OS) of 10.1 months [52]. A separate phase 2 trial of lenvatinib in patients with *FGFR*-mutated endometrial cancer reported ORRs of 14.3–21.8%, PFS of 5.4 months, and OS of 10.6 months [53]. In addition, the ROCOCO phase 1 study is studying the combination of rogaratinib with copanlisib in patients with solid tumors complicated by *FGFR1–4* mutations [54]. Taken together, therapy targeting FGFR3 mutation may improve the outcomes of therapy for endometrial CCC. Thus, the cell line can be used for developing methods for early diagnosis, for investigating new targeting therapy including *ARID1A* and *FGFR3*, and for examining the involvement of *ARID1A* and *FGFR3* mutation in the development, survival, and progression of cancer.

## Conclusion

In conclusion, we successfully derived a unique endometrial CCC cell line from a patient for use in future cancer research. Specifically, the cell line can be used for further endometrial CCC research to clarify the pathogenic mechanism and improve treatment.

## Supplementary Information

**Additional file 1: Supplement Table 1.** Profiling of short tandem repeat in 150,057 cells and DNA, and the comparison of the ATCC STR database.

**Additional file 2:.**

## Data Availability

The datasets used and/or analysed during the current study available from the corresponding author on reasonable request.

## Abbreviations

CCC: Clear cell carcinoma
*FGFR3*: *Fibroblast growth factor receptor 3*
*ARID1A*: *AT-Rich Interaction Domain 1A*
FIGO: International Federation of Gynecology and Obstetrics
IRB: Institutional review board
PBS: Phosphate-buffered saline
UV: Ultraviolet
H&E: Hematoxylin and eosin
WT1: Wilms’ tumor 1
HNF1β: Human nuclear factor 1
ER: Estrogen receptor
PR: Progesterone receptor
HRP: Horseradish peroxidase
DAB: Diaminobenzidine tetrahydrochloride
DMEM: Dulbecco’s Modified Eagle’s Medium
ATCC: American Type Culture Collection
STR: Short tandem repeats
CK: Cytokeratin
ELISA: Enzyme-linked immunosorbent assay
XTT: 2H-tetrazolium, 2,3-bis (2-methoxy-4-nitro-5-sulfophenyl)-5-[(phenylamino)carbonyl]-hydroxide
CSC: Cancer stem cell
IC50: Half-maximal inhibitory concentrations
NOD-SCID: Non-obese diabetic-severe combined immunodeficiency
SNP: Single nucleotide polymorphism
CCr: Creatinine clearance
MAPK: Mitogen-activated protein kinase
PI3K/AKT: Phosphoinositide 3-kinases/ Protein kinase B
PLCγ: Phospholipase C-γ
STAT: Signal transducer and activator of transcription
SWI/SNF: Switch/sucrose non-fermentable
