# Supplementary material for: A clear cancer cell line (150057) derived from human endometrial carcinoma harbors two novel mutations
Source: BMC Cancer. 2020 Nov 3;20:1058. doi: 10.1186/s12885-020-07567-w (PMC7607743; doi:10.1186/s12885-020-07567-w)
Supplement: Supplementary file 1 — Additional file 1: Supplement Table 1. Profiling of short tandem repeat in 150,057 cells and DNA, and the comparison of the ATCC STR database. [file 12885_2020_7567_MOESM1_ESM.docx]

### **Supplement Table 1. Profiling of short tandem repeat in 150057 cells and DNA, and the comparison of the ATCC STR database.**

| Sample | TH01 | D5S818 | D13S317 | D7S820 | D16S539 | CSF1PO | AMEL | vWA | TPOX | Match% |
| --- | --- | --- | --- | --- | --- | --- | --- | --- | --- | --- |
| 150057 cells | 7,9 | 11 | 8,10 | 10,11 | 9,13 | 9,11 | X | 16,17 | 11 | - |
| 150057 DNA | 7,9 | 11 | 8,10 | 10,11 | 9,13 | 9,11 | X | 16,17 | 11 | 100 |
| ATCC STR result | There are no results | | | | | | | | | |

### STR: short tandem repeat
