# Supplementary material for: A clear cancer cell line (150057) derived from human endometrial carcinoma harbors two novel mutations
Source: BMC Cancer. 2020 Nov 3;20:1058. doi: 10.1186/s12885-020-07567-w (PMC7607743; doi:10.1186/s12885-020-07567-w)
Supplement: Supplementary file 2 — Additional file 2:. [file 12885_2020_7567_MOESM2_ESM.pdf]

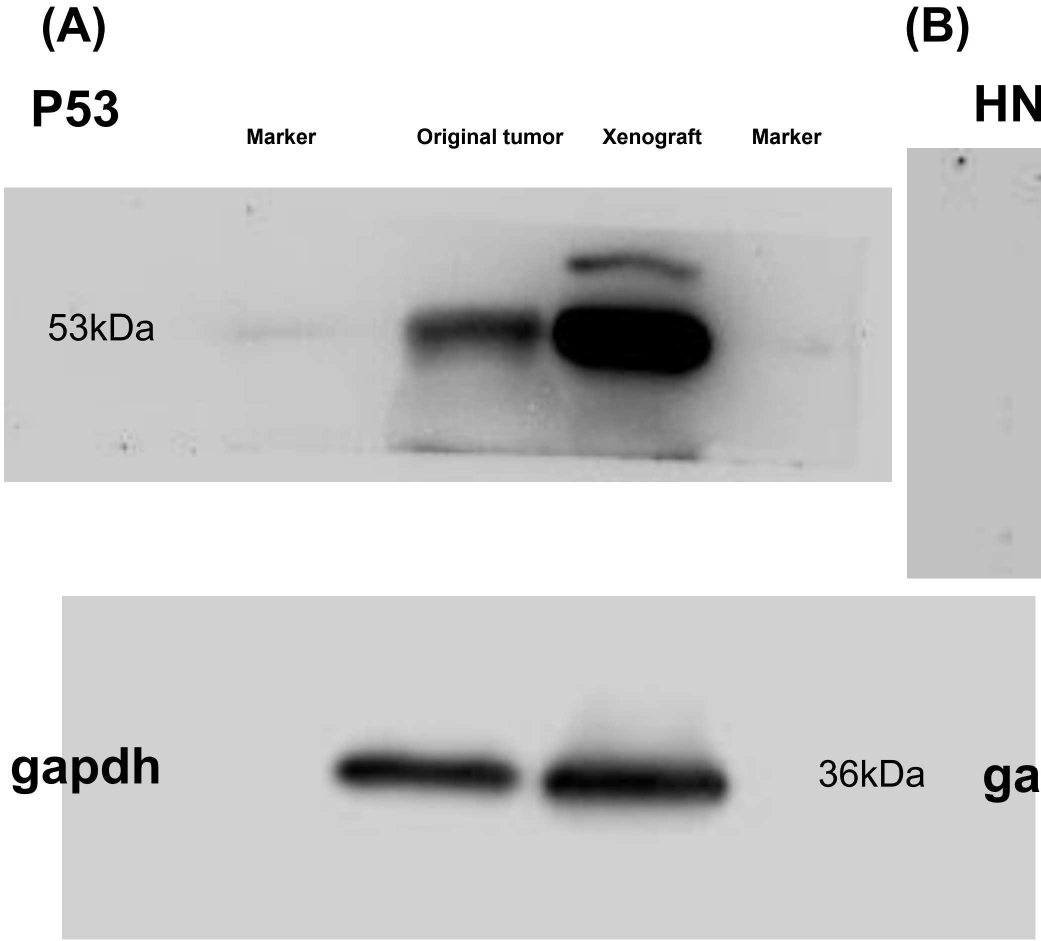
(A)
P53
Marker
Original tumor
Xenograft
Marker
53kDa
gapdh
36kDa

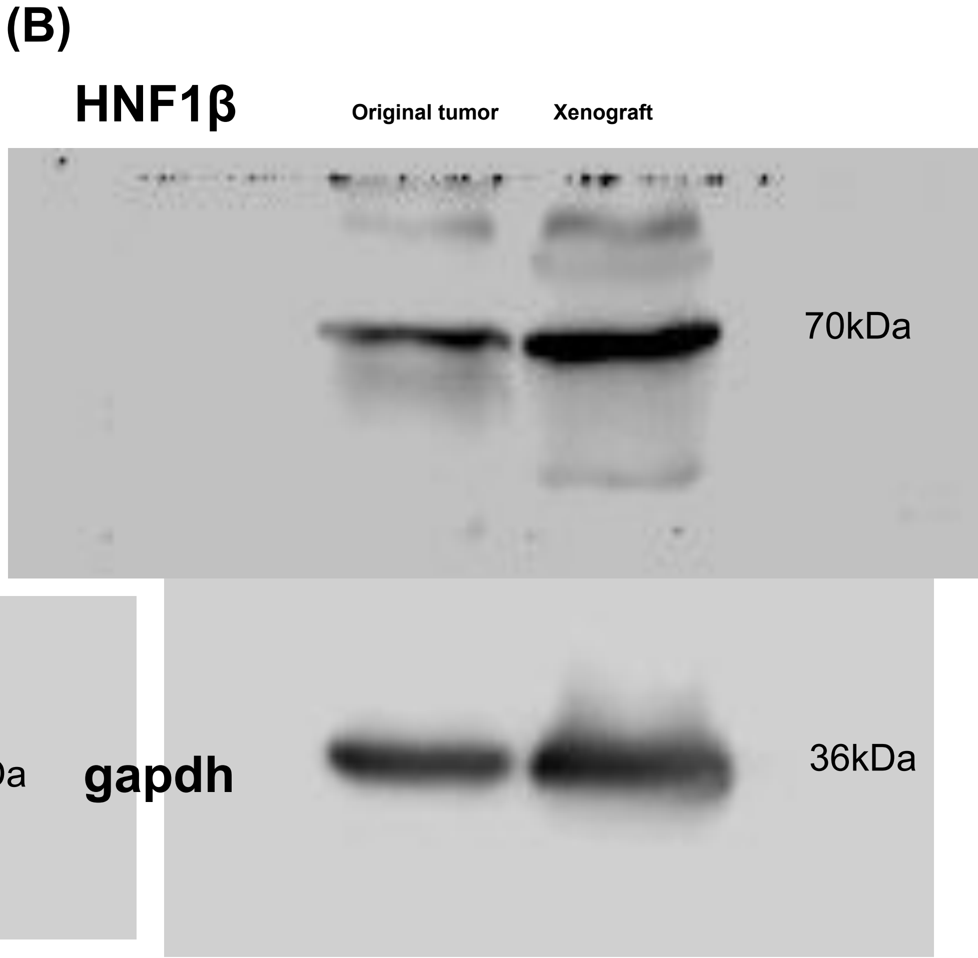
(B)
HNF1β
Original tumor
Xenograft
70kDa
gapdh
36kDa
